# Placenta Accreta Spectrum Outcomes Using Tourniquet and Forceps for Vascular Control

**DOI:** 10.3389/fmed.2021.557678

**Published:** 2021-10-18

**Authors:** Jingrui Huang, Xiaowen Zhang, Lijuan Liu, Si Duan, Chenlin Pei, Yanhua Zhao, Rong Liu, Weinan Wang, Yu Jian, Yuelan Liu, Hui Liu, Xinhua Wu, Weishe Zhang

**Affiliations:** ^1^Department of Obstetrics, Xiangya Hospital Central South University, Changsha, China; ^2^Department of Radiology, Xiangya Hospital Central South University, Changsha, China; ^3^Hunan Engineering Research Center of Early Life Development and Disease Prevention, Changsha, China

**Keywords:** classification, placenta accreta spectrum, pregnancy outcome, cesarean section, surgery

## Abstract

**Objective:** To evaluate the use of tourniquet and forceps to reduce bleeding during surgical treatment of severe placenta accreta spectrum (placenta increta and placenta percreta).

**Methods:** A tourniquet was used in the lower part of the uterus during surgical treatment of severe placenta accreta spectrum. Severe placenta accreta spectrum was classified into two types according to the relative position of the placenta and tourniquet during surgery: upper-tourniquet type, in which the entire placenta was above the tourniquet, and lower-tourniquet type, in which part or all of the placenta was below the tourniquet. The surgical effects of the two types were retrospectively compared. We then added forceps to the lower-tourniquet group to achieve further bleeding reduction. Finally, the surgical effects of the two types were prospectively compared.

**Results:** During the retrospective phase, patients in the lower-tourniquet group experienced more severe symptoms than did patients in the upper-tourniquet group, based on mean intraoperative blood loss (upper-tourniquet group 787.5 ml, lower-tourniquet group 1434.4 ml) intensive care unit admission rate (upper-tourniquet group 1.0%, lower-tourniquet group 33.3%), and length of hospital stay (upper-tourniquet group 10.2d, lower-tourniquet group 12.1d). During the prospective phase, after introduction of the revised surgical method involving forceps (in the lower-tourniquet group), the lower-tourniquet group exhibited improvements in the above indicators (intraoperative average blood loss 722.9 ml, intensive care unit admission rate 4.3%, hospital stays 9.0d). No increase in the rate of complications was observed.

**Conclusion:** The relative positions of the placenta and tourniquet may influence the perioperative risk of severe placenta accreta spectrum. The method using a tourniquet (and forceps if necessary) can improve the surgical effect in cases of severe placenta accreta spectrum.

## Introduction

Placenta accreta spectrum (PAS) includes adherent placenta accreta (placenta creta, vera or adherenta), placenta increta, and placenta percreta (1). Placenta previa and previous cesarean delivery are the two most important risk factors for PAS (2). When these factors are present, cesarean scar pregnancy can occur during early pregnancy, whereas PAS most commonly occurs in the third trimester (3, 4). The greatest challenge in managing this spectrum of complications involves controlling antepartum and intraoperative hemorrhage. Severe hemorrhage is an important cause of PAS-related hysterectomy, disseminated intravascular coagulation, and maternal mortality (2, 5). Because almost all pregnant women with PAS undergo planned cesarean delivery, controlling intraoperative bleeding is important for the management of PAS.

An optimal international standard for PAS surgery is not yet available, and the establishment of a suitable surgical method is an important focus of in PAS management. Therefore, obstetricians have adopted various approaches to optimize cesarean section (3, 6–8). These methods can reduce blood loss and improve patient prognosis. However, in some cases of severe PAS, the placenta is implanted into the pelvic cavity with an abundant blood supply; thus, peripartum hemorrhage (and even hysterectomy) may still occur (2, 9, 10).

Internal iliac artery occlusion or similar interventions can reduce bleeding, but side effects include iliac or femoral artery thrombosis or rupture, as well as ischemic nerve injury. Considering the need for multidisciplinary cooperation, these may become a limitation of such occlusion treatment methods (11). Because most PAS patients undergo cesarean sections, improvements in surgical methods and the reduction of bleeding remain important.

Placental blood supply is the main cause of intraoperative bleeding (12–15). Differences in placental position and the degree of implantation may require distinct strategies to control intraoperative bleeding. Tourniquets have been used in PAS to reduce intraoperative bleeding by blocking or reducing blood flow to the placenta (16–18). However, the position of the placenta may be particularly problematic in cases of severe PAS, involving scenarios such as invasion of the cervix. The effectiveness of tourniquets to reduce this type of bleeding must be reevaluated. Therefore, the present study was performed based on the relative positions of the tourniquet and placenta, with the aim of designing an improved surgical strategy for PAS. First, we placed a tourniquet in the lower part of the uterus, which was presumed to partially block the placental blood supply. PAS can also be classified according to the relative positions of the tourniquet and the placenta. Lower placental position may be associated with higher surgical risk. The surgical effects of tourniquets were also studied. To reduce bleeding in cases of lower placenta accreta, we added forceps to block the blood flow to the lower placenta. If tourniquets and forceps can effectively reduce intraoperative bleeding in PAS surgery, the surgery (effects) could be improved.

## Methods

### Clinical Data

**Figure 1:**
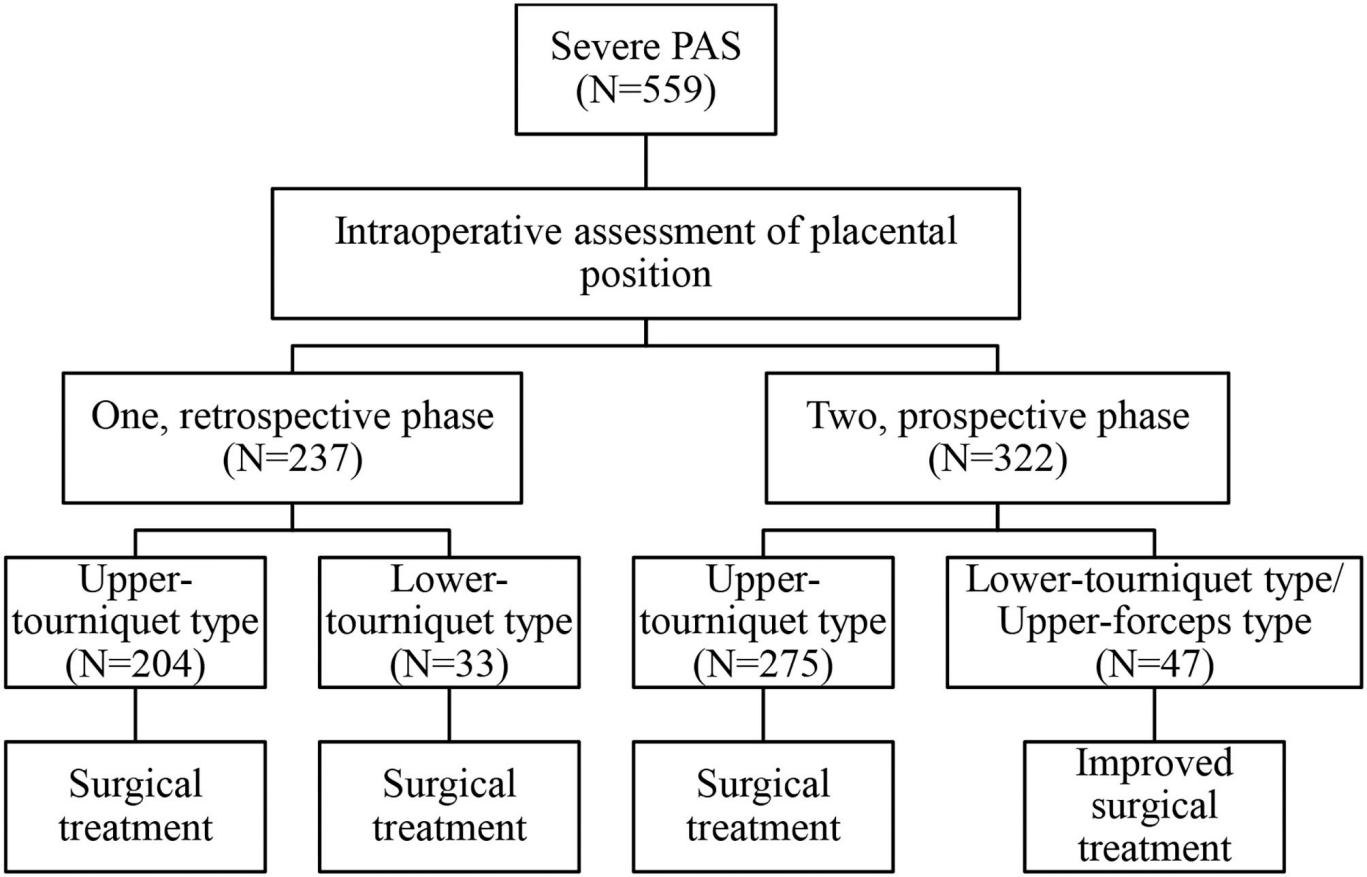
Flow chart of the study. PAS, placenta accreta spectrum.

The study included 559 severe PAS patients treated at Xiangya Hospital Central South University, that were followed for 3 months after delivery. Postoperative follow-up included symptoms (e.g., infection, fever, dizziness, urination, abnormal vaginal bleeding, and abdominal pain) and examinations (e.g., routine blood tests, C-reactive protein, routine urinalysis, and ultrasound). This study compared different case types and surgical methods (Figure 1). According to the admission date and surgical classification, 237 cases from January 1, 2016 to December 31, 2017 were classified as retrospective group. Three hundred twenty-two cases from January 1, 2018 to December 31, 2019 were classified as prospective group.

In general, retrospective data were used to assess the intraoperative classification process and the risks associated with different PAS types. Prospective data were used to evaluate the effectiveness of surgical method selection based on the intraoperative classification. The detailed classification and intraoperative measures are described in the following sections.

### Inclusion Criteria

**Figure 2:**
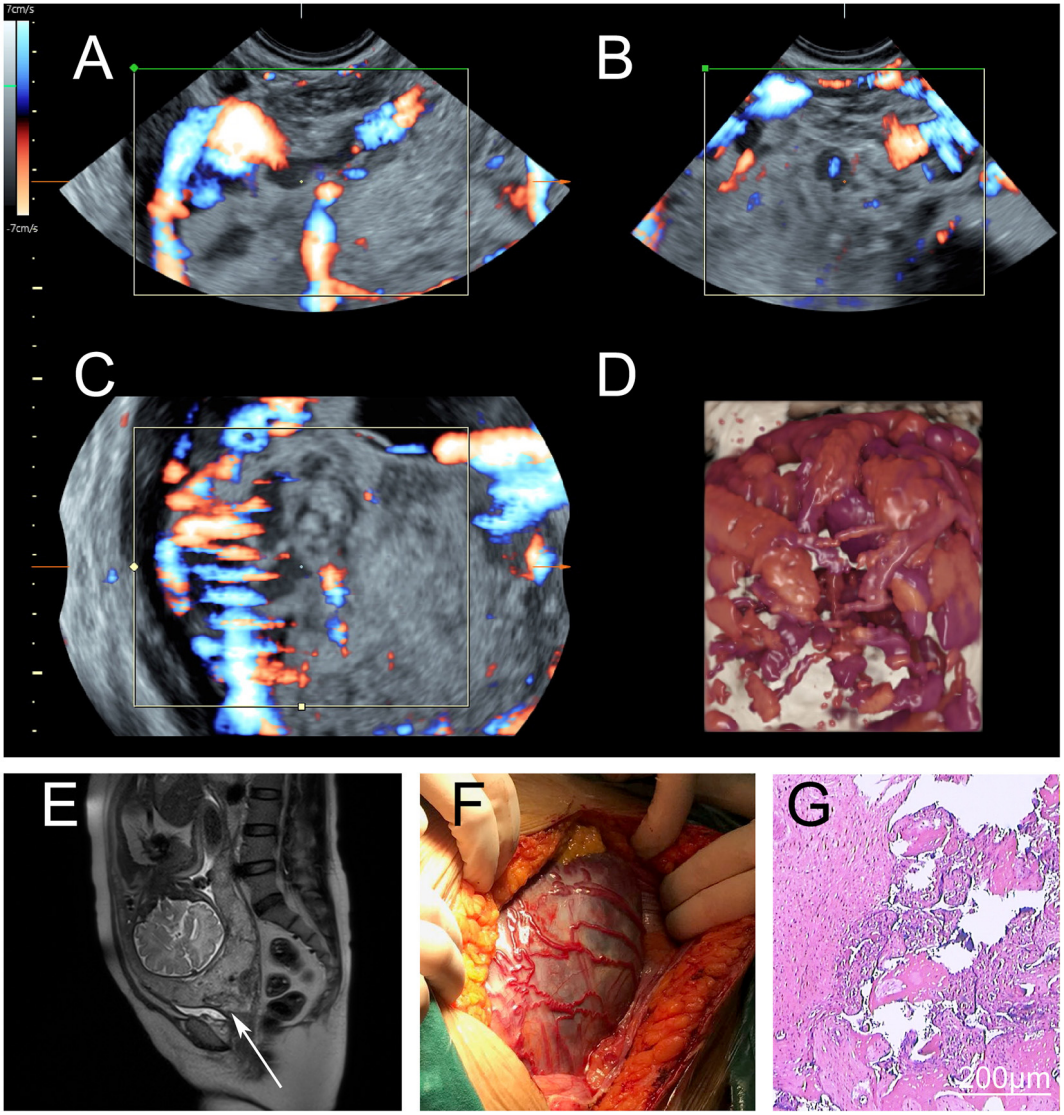
Perioperative imaging of severe placenta accreta spectrum. **(A–D)** Preoperative magnetic resonance imaging of a severe placenta accreta spectrum patient. The placenta had invaded the myometrium and formed a large number of irregular blood vessels. During the operation, the deformed blood vessels were found under the serosa between the uterus and bladder. **(A–C)** Images of the different sections and blood flow. **(D)** Three-dimensional simulation image. **(E)** Preoperative magnetic resonance imaging. The cervix was invaded by the placenta (arrow). **(F)** Intraoperative observations. There were many dilated blood vessels in the lower uterus, and the placenta had invaded the serosa. **(G)** Postoperative pathological examination. The myometrium was invaded by trophoblasts.

All patients were diagnosed using ultrasound or magnetic resonance imaging (Figure 2). Preoperative imaging examination, intraoperative observation, and postoperative pathology were used to diagnose placenta increta or placenta percreta. The exclusion criteria were: (1) gestational age <28 weeks; (2) stillbirth; (3) multiple pregnancies; (4) hematological diseases: primary coagulation dysfunction, thrombocytopenia, aplastic anemia, et al.; (5) severe pregnancy complications and comorbidities, malformed uterus, systematic wasting disease, serious conglutination of pelvic cavity and bowel; and (6) emergency surgery for hemorrhagic shock that we were unable to assess in this study.

### Intraoperative Classification and Improved Surgical Methods

In accordance with the standards for cesarean section procedures used in PAS (19), a 13-cm incision was made; the length of the incision was adjusted according to the level of exposure required. After the adhesion had been separated, the fetus was removed by avoiding the main part of the placenta.

After fetal delivery, the uterus was extracted from the abdominal cavity and pulled upward to make space for the tourniquet (rubber band, No.8). The assistant's right hand reached into the rectouterine pouch and lifted the cervix. The tourniquet was slid down (caudal) near the outside of the assistant's hand to the lowest point, fixed by the assistant, and then tightened using long curved forceps from the front of the uterus. Finally, the tourniquet was fixed above the level of the uterosacral ligament to stop bleeding. Here, if the placenta was entirely above the tourniquet, this was defined as the upper-tourniquet type; otherwise, it was considered the lower-tourniquet type, in which placental tissue was present under the tourniquet (only a tourniquet was used in all cases in the retrospective analysis).

**Figure 3:**
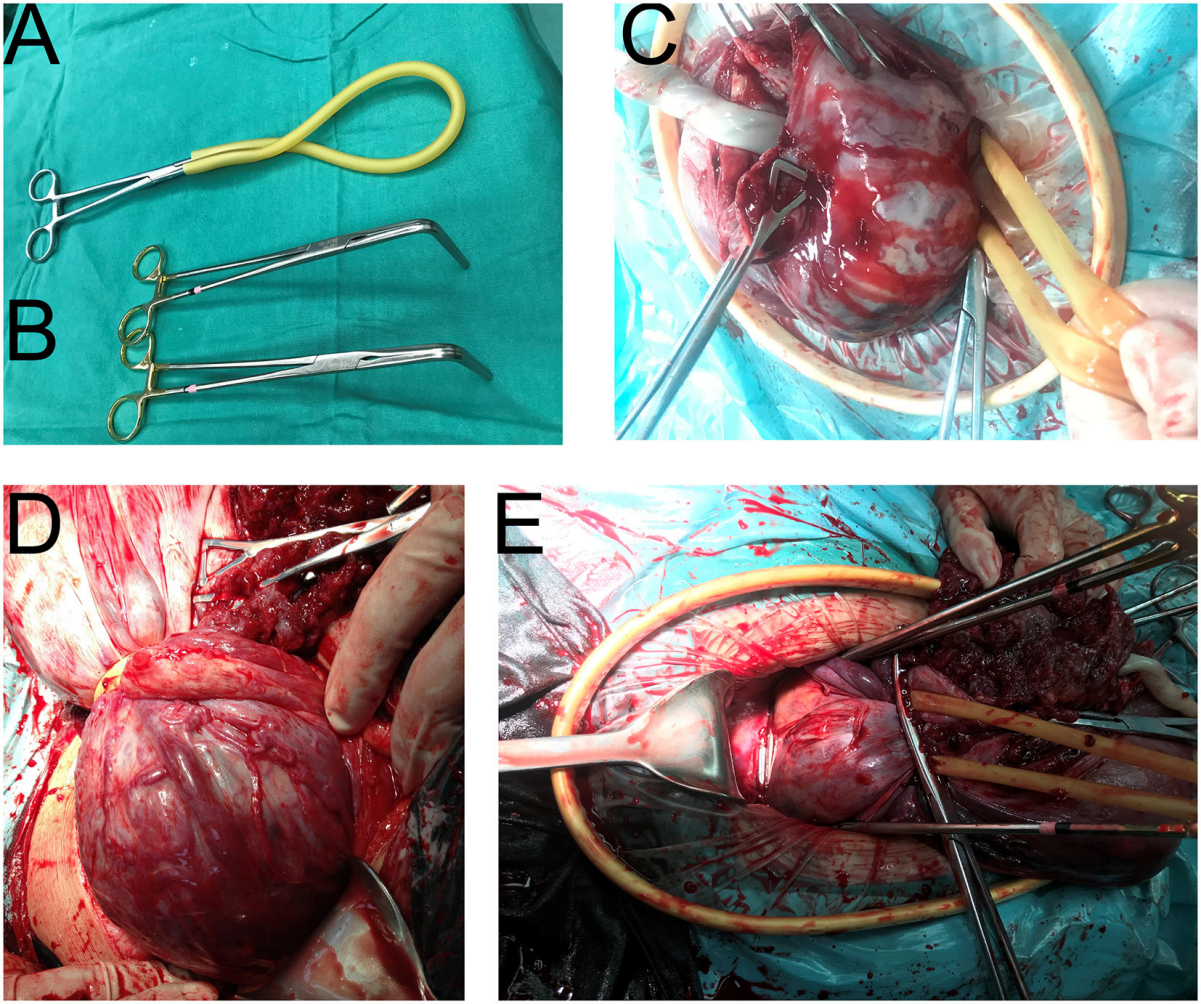
Intraoperative application of our special surgical instruments. **(A)** The tourniquet (rubber band, No.8). **(B)** Two bronchus forceps, 15 cm. **(C)** The upper-tourniquet type. All placenta was above the tourniquet. **(D)** The lower-tourniquet type. Some of the placenta was below the tourniquet. **(E)** The upper-forceps type. After the application of two forceps, the lower-tourniquet type was converted to the upper-forceps type. The forceps were used to support the placenca and block the blood flow.

If placental tissue was present under the tourniquet, the avascular area of the posterior peritoneum and broad ligament was opened; this was followed by forceps (non-invasive bronchus forceps, 15 cm) insertion along the pelvic wall. Under the placental tissue (at the level of the external cervix and top of the vagina), the two-sided forceps were clamped to block the relevant blood supply. Here, the lower-tourniquet type was now considered the upper-forceps type, following improvements with tourniquet and forceps to generate the lower-tourniquet/upper-forceps type (prospective analysis only). The absence of placenta under the forceps was confirmed through exploration, and the assistant confirmed the absence of vaginal bleeding (Figure 3, Supplementary Figure 1, Supplementary Video 1).

After bleeding had been controlled, the placenta was peeled off until complete separation. If a portion of the placenta penetrated the uterine serous membrane, local excision, and repair surgery were performed. The myometrium was then sutured to stop bleeding. The external cervix (i.e., top of the vagina) was lifted above the forceps, and the cervix and lower part of the uterus were reconstructed. If severe placenta percreta was evident in the lower uterus, a lower uterine segment constriction suture or uterine B-Lynch suture procedure was performed. If massive bleeding occurred after surgery, arterial embolization was considered for hemostasis. All placentas and removed tissues from the placental implantation site were sent for pathological examination.

### Statistical Analysis

The statistical analysis was performed by SPSS 22.0 software, the measurement data were expressed as χ±s, and the count data were expressed as n (%). Statistical methods are listed after all tables. *P* < 0.05 was considered as a statistically significant difference.

## Results

### General Information

There were 237 retrospective cases (237/559, 42.4%) and 322 prospective cases (322/559, 57.6%). There were no significant differences in the general characteristics of the two groups; thus, subjects in the two groups were comparable (Table 1). The most accurate diagnosis of PAS is achieved by pathological examination after hysterectomy. However, all of our patients had a strong desire to preserve the uterus; because of our surgical strategy, there were almost no cases of hysterectomy. Considering the possibility of inconsistencies among preoperative, surgical, and pathological diagnoses, all placentas and removed tissues from the placental implantation site were sent for pathological examination. The results confirmed the presence of PAS, consistent with preoperative and intraoperative diagnoses. The surgical method was determined based on the intraoperative diagnosis, and the final diagnosis was made on the basis of intraoperative conditions and pathological diagnosis. Different subtypes were distinguished mainly on the basis of intraoperative conditions and pathological diagnosis. To describe the intraoperative diagnosis more accurately, we classified PAS (Table 2) according to the International Federation of Gynecology and Obstetrics (FIGO) guidelines (all cases were classified as grade 3, 4, 5, or 6), which is related to the three subtypes including placenta creta, vera or adherenta; placenta increta; and placenta percreta (1, 20).

**Table 1:** General characteristics of cases in retrospective and prospective analyses.

**Information**	**Retrospective data**	**Prospective data**	***P*-value**
Types *N* (%)	237 (100)	322 (100)	0.823^*^
Upper-tourniquet type, *N* (%)	204 (86.1)	275 (85.4)	–
Lower-tourniquet type or upper-forceps type, *N* (%)	33 (13.9)	47 (14.6)	–
Age (y)	32.6 ± 4.3	33.2 ± 3.5	0.590
Gravidity (times)	4.0 ± 1.9	4.4 ± 1.6	0.070
Parity (times)	2.0 ± 0.6	2.6 ± 0.9	0.502
Previous abortion (times)	1.8 ± 1.4	1.8 ± 1.5	0.474
Previous cesarean (times)	1.7 ± 0.6	1.8 ± 1.2	0.514
Interval between previous cesarean (y)	4.3 ± 4.6	4.4 ± 3.8	0.212
BMI (kg/m^2^)	24.6 ± 5.8	25.2 ± 6.8	0.345
Preoperative HGB (g/l)	103 ± 3.8	104 ± 2.9	0.881
Gestational week (weeks)	36.2 ± 1.8	36.1 ± 2.1	0.628
Birth weight (g)	2723.2 ± 517.1	2702.5 ± 438.7	0.865
Apgar 1 min (score)	9.1 ± 1.7	9.0 ± 1.4	0.164
Apgar 5 min (score)	9.6 ± 1.1	9.5 ± 1.1	0.563

* *χ^2^-test. Others: student's t-test. BMI, body mass index; HGB, hemoglobin*.

**Table 2:** Clinical classifications of cases in retrospective and prospective analyses.

**Clinical classification grade**	**Retrospective data**	**Prospective data**	*P*-value^*^
Total *N* (%)	237 (100)	322 (100)	0.387
Grade 3, *N* (%)	71 (30.0)	105 (32.6)	–
Grade 4, *N* (%)	66 (27.8)	99 (30.7)	–
Grade 5, *N* (%)	65 (27.4)	68 (21.1)	–
Grade 6, *N* (%)	35 (14.8)	50 (15.5)	–

* *χ^2^-test*.

### Perioperative Information

Based on comparisons of perioperative indicators, we found that lower-tourniquet cases were more severe than upper-tourniquet cases (Table 3). Similarly, analysis of retrospective data indicated that PAS cases could be classified intraoperatively into two types with different risks and outcomes.

**Table 3:** Comparison of different types of perioperative indicators.

**Groups/Variable**	**Upper-tourniquet type**	**Lower-tourniquet type/Upper-forceps type**	***P*-value**
**Total cases**, ***N***	**479**	**80**	**–**
Intraoperative blood loss (ml)	734.1 ± 489.4	1283.4 ± 826.1	0.009
RBC infusion (U)	0.54 ± 2.38	1.49 ± 2.81	0.031
Autologous blood transfusion (ml)	169.4 ± 87.8	243.3 ± 91.5	0.008
ICU admission, *n* (%)	3 (0.6)	13 (16.3)	0.000^*^
Hospital stays (d)	9.2 ± 3.1	11.1 ± 2.9	0.042
Hospitalization expenses (10 thousand Yuan)	2.0 ± 0.8	3.4 ± 1.3	0.039
**Retrospective group**, ***N***	**204**	**33**	**–**
Intraoperative blood loss (ml)	787.5 ± 501.7	1434.4 ± 902.2	0.000
RBC infusion (U)	0.6 ± 2.7	2.4 ± 4.1	0.000
Autologous blood transfusion (ml)	173.0 ± 90.3	314.3 ± 83.8	0.006
ICU admission, *n* (%)	2 (1.0)	11 (33.3)	0.000^*^
Hospital stays (d)	10.2 ± 3.1	12.1 ± 4.7	0.038
Hospitalization expenses (10 thousand Yuan)	1.6 ± 1.2	2.0 ± 2.6	0.043
**Prospective group**, ***N***	**275**	**47**	**–**
Intraoperative blood loss (ml)	682.1 ± 521.7	722.9 ± 602.2	0.118
RBC infusion (U)	0.5 ± 1.4	0.6 ± 1.3	0.234
Autologous blood transfusion (ml)	168.7 ± 85.3	184.3 ± 99.8	0.286
ICU admission, *n* (%)	1 (0.4)	2 (4.3)	0.057^**^
Hospital stays (d)	8.6 ± 3.5	9.0 ± 2.7	0.136
Hospitalization expenses (10 thousand Yuan)	1.5 ± 1.3	1.7 ± 1.8	0.183

* *Continuity correction χ^2^-test*,

** *Fisher's exact probability test, others: t test. RBC, red blood cell; ICU, intensive care unit*.

During the prospective phase, the surgical method used in the upper-tourniquet cases was unchanged, whereas the upper-forceps method was used in lower-tourniquet cases. Our analysis of prospective data revealed that patient outcomes did not significantly differ between the upper-tourniquet and lower-tourniquet groups. These observations indicated that the change in surgical method for lower-tourniquet cases (i.e., switching to the upper-forceps method) led to improvements in perioperative indicators including intraoperative blood loss, volume of red blood cell infusion required, amount of autologous blood transfusion required, and length of hospital stay.

### Effects of Surgical Method Revision on Hysterectomy and Complication Rates

No patients experienced severe bleeding during and after surgery; thus, embolization and similar interventional treatments were not performed. Table 4 describes the long-term complications observed during postoperative follow-up, including late postpartum hemorrhage, puerperal infection, and urinary fistula. No abnormalities were found in the remaining patients (in Table 4) at 3 months postoperatively. Overall analysis showed that the hysterectomy rate was significantly lower in the prospective group than in the retrospective group, but there were no significant differences in complications between the two groups. Furthermore, the short-term complications and hysterectomy rates were significantly lower in the upper-forceps type in the prospective data, compared with the lower-tourniquet type in the retrospective group. These findings suggested that our improved surgical procedures reduced the hysterectomy and short-term complication rates without increasing the long-term complication rate (Table 4).

**Table 4:** Comparison of different surgical methods of complications.

**Complications, *N* (%)**	**Retrospective group**	**Prospective group**	***P*-value**
**All types**	**237**	**322**	**–**
Short-term complications	11 (4.6)	7 (2.2)	0.102
Long-term complications	4 (1.7)	1 (0.3)	0.210^*^
Readmission	3 (1.3)	1 (0.3)	0.414^*^
Hysterectomy	8 (3.4)	1 (0.3)	0.012^*^
**Upper-tourniquet type**	**204**	**275**	**–**
Short-term complications	2 (0.8)	3 (0.9)	1.000^*^
Long-term complications	0	0	–
Readmission	0	0	–
Hysterectomy	0	0	–
**Lower-tourniquet type or upper-forceps type**	**33**	**47**	**–**
Short-term complications	9 (27.3)	4 (8.5)	0.025
Long-term complications	4 (12.1)	1 (2.1)	0.177^*^
Readmission	3 (9.1)	1 (2.1)	0.376^*^
Hysterectomy	8 (24.2)	1 (2.1)	0.006^*^

* *Continuity correction χ^2^-test. Others: χ^2^-test. Short-term complication: intraoperative injuries of the iliac vessels, bladder or ureters, paralytic ileus, and thrombogenesis. Long-term complications: late postpartum hemorrhage, puerperal infection, and urinary fistula*.

## Discussion

The main findings of the present study were as follows: (1) severe PAS can be classified based on the relative position of the placenta during surgery, and different PAS types are associated with distinct risks of intraoperative hemorrhage; (2) PAS with deeper placental invasion, a lower placenta, and a placenta that cannot be fixed by a tourniquet during surgery is associated with higher risk; (3) a revised surgical method based on the classification results can improve clinical outcomes in severe PAS, particularly in cases with lower placental implantation, and there was no evidence that this surgical method resulted in higher complication rates.

Satisfactory surgical outcomes can be achieved in mild PAS (6, 7, 21). However, severe PAS is associated with high risks of postpartum hemorrhage and hysterectomy, particularly if the placenta invades the pelvic organs (e.g., cervix or bladder). The treatment is generally based on placental separation and bleeding control depending on the degree of implantation. Treatment can include preservation of the placenta *in situ* or hysterectomy (with or without the placenta) (6, 8, 22).

The limitations of these treatments may include long hospital stays, infertility, and postoperative complications. For cases involving retention of the placenta, follow-up is ~6 months, which increases the risks of infection and bleeding (delayed bleeding and endometritis are the main complications) (7). Hysterectomy is a major surgery that leads to the loss of future fertility (23, 24). However, hysterectomy is an important treatment option for uncontrolled bleeding (25, 26). Interventional radiological methods (such as uterine arterial embolization) can significantly reduce bleeding. However, medical complications (e.g., renal failure, infection, and rupture of pseudoaneurysms) may limit their use (6, 15, 27). In comparison, our surgical options do not include interventional methods, thereby reducing the risks of related complications. Blocking the blood flow during surgery is a more direct approach, which helps to improve visualization and operability.

Obstetricians commonly use several methods of hemostasis, including abdominal aortic balloons and uterine arterial embolization (15, 28–31). These approaches may be limited by the need for multiple interventions and the risk of complications. Furthermore, these procedures may require multidisciplinary cooperation, as well as indwelling arterial catheters, thus increasing the complication risks and medical costs. In some cases, particularly when interventional surgery is not possible, the use of direct hemostasis during a cesarean section is considered.

Because each of our patients had a strong desire to preserve the uterus, surgical approaches to preserve the uterus were considered as the main treatment options. The improved surgical methods also focused on preserving the uterus and reducing bleeding. Our general approach involves first blocking or reducing the blood supply to the placenta through the use of a tourniquet and/or forceps. If bleeding can be controlled, a comprehensive hemostasis strategy is required. The placenta is then removed. If a portion of the placental implantation site penetrates the serosal layer, local excision, and repair surgery are performed. Tourniquets or forceps are components of a bleeding reduction strategy. After combined intraoperative hemostasis in all patients, we observed no cases of severe bleeding after surgery; thus, radiological hemostasis was not performed. Upon return to the ward after surgery, radiological hemostasis was considered in cases that continued to exhibit a large amount of bleeding. Therefore, with the goal of preserving the uterus, we attempted to reduce bleeding during surgery and remove the placenta using our improved surgical hemostasis methods; other supplemental hemostasis methods were used after surgery. Importantly, the detailed PAS surgical methods should be based on the global PAS treatment framework; all methods should be determined in a patient-specific manner.

The main PAS surgical methods are reported in the FIGO guidelines, which including conservative management (manual removal of the placenta, leaving the placenta *in situ* or the expectant approach, removal of the accreta area, and suturing around the accreta area after resection) and non-conservative surgery (cesarean hysterectomy) (32, 33). Some studies have reported good effects with respect to the use of tourniquets in PAS surgery (16–18). The use of tourniquets is flexible and variable. A recent study used bilateral tourniquets placed on the upper uterine pedicles and on the cervicoisthmic segment, as well as controlled Zhukovsky balloon tamponade of the uterus (34). These methods produced a mean blood loss volume of 1,295 ± 520.3 ml (*n* = 21). The studies thus far have suggested that tourniquets can reduce blood supply to the placenta during surgery, thus reducing intraoperative bleeding in PAS. However, the tourniquets for reducing blood supply to the placenta may be insufficient for some cases of severe PAS. Furthermore, severe placenta percreta near the cervix was occasionally located below the tourniquet. Therefore, we added forceps under the tourniquet to block the blood supply to the placenta.

As a radiological method, subrenal abdominal aortic balloon occlusion can be used for PAS, with a mean blood loss volume of 1600.00 ± 1185.785 ml (35). The abdominal aorta should not be occluded for an excessive duration, and clinical guidelines recommend that each occlusion time should not exceed 60 min (35). A recent randomized controlled trial suggested that intraoperative balloon occlusion of the internal iliac arteries did not reduce the number of packed red blood cell units transfused in women with placenta previa and antenatally suspected placenta accreta (36). Therefore, in addition to optimizing surgical procedures, further evidence is needed to evaluate radiological methods, such as balloon occlusion (37).

The placental blood supply is an important factor that affects intraoperative bleeding and hysterectomy rates (38, 39). During the retrospective phase, upper-tourniquet patients experienced a significant reduction in blood loss because of the blocked blood supply, and no hysterectomy was performed. In contrast, severe bleeding and hysterectomy occurred in lower-tourniquet patients, although this group comprised only 14% of the patients. During the prospective phase of the study, we also found that intraoperative bleeding in lower-tourniquet patients significantly decreased when the upper-forceps method was used, because the blood flow was blocked using this method. The blocking location is closely associated with the hemostatic effect. Therefore, the use of an upper-forceps method in lower-tourniquet cases is an important measure to reduce bleeding and improve surgical outcomes. The main reason for non-fixation of the occluding tourniquet below the cervix was that the elastic occlusion tourniquet could slide into the uterine isthmus because of the tension and pulling force exerted by the tissue. In contrast, noninvasive hemostatic forceps and bronchus forceps are not elastic; thus, they are not easily dislodged. For a placenta located in the deep pelvic cavity and above the cervix, these surgical instruments can be used to control bleeding. For convenience, two bronchus forceps can be used to create a surgical pack in cases of severe PAS.

Marked by the uterosacral ligament, the tourniquet is adjacent to the upper part of the uterosacral ligament (i.e., the isthmus uteri or lower part of uterus). This affects the placental blood supply from the ascending branch of the uterine artery, arcuate artery, and spiral artery; it reduces blood flow in this region (40–43). Notably, this is also the main blood supply to the upper part of the uterus. In contrast to the upper part of the uterus, the blood vessel network of the lower part of the uterus is mainly composed of three main arteries: the descending branch of the uterine artery, the cervical artery, and the vaginal artery (40–43). There are distinct types of traffic anastomosis among the three arteries. In some severe cases, the placenta remains under the tourniquet (e.g., when the placenta invades the cervix). The forceps were added at the level of external cervix (i.e., top of the vagina) to block blood supply; this affected the cervicovaginal branch of the uterine artery, the cervical artery, and the ascending branch of the vaginal artery. Approximately 67% of blood supply of the cervical artery originates from the uterine artery, 23% originates from the vaginal artery, and 10% originates from the lower vesical artery (43). Therefore, the blood supply differs among placental implantation positions. Based on these differences, tourniquets and forceps can block most of the blood supply to the placenta, thereby reducing intraoperative bleeding. However, because of the blood supply complexity in PAS, further detailed research will help to clarify the sources of placental blood supply. The various effects of our hemostatic measures illustrate the relationship between the placental position and the blood supply. The lower placenta may have a more abundant blood supply. This could help explain the uncertain effects of vascular embolization and intraoperative vascular blockade. Our intraoperative classification scheme can be used as a reference for assessing the risk of intraoperative bleeding, as well as for surgical planning.

The anterior wall (placenta) is next to the bladder; it exhibits distinct strength and toughness, compared with arterial or bronchial tissue. We also evaluated the risk of tissue damage from this procedure. With the exception of the effects of surgery, no significant differences were observed in the incidences of short-term and long-term complications according to the procedure, indicating that there were no increased risks of complications.

Our study evaluated the outcomes in case of severe PAS when the surgical method had been revised based on the results of intraoperative classification. Hysterectomy may be necessary in some cases of severe PAS, suggesting that other factors (i.e., in addition to placental location) may affect the treatment outcome. However, our data did not permit the assessment of these potential factors. In addition, our study excluded complications associated with interventional methods; future studies should focus on the incidences of long-term complications associated with surgery, such as the success rates of subsequent pregnancies.

Although we did not observe increased complication rates, further studies should be performed to explore rare complications. Moreover, because this was a single-center study, there is a need to consider the possibility of selection bias. Our research was mainly based on the position of the placenta and the relative position of the hemostatic device. If both the localized and diffuse types are located above the tourniquet, the tourniquet is mainly used for hemostasis; otherwise, the tourniquet and forceps are used to stop bleeding. Future research could explore the abovementioned types and their surgical options. Finally, this study focused on the procedures employed during cesarean section, which require advanced surgical skills. It is important for the obstetrician to assess the condition of each patient and prepare a personalized surgical plan.

In summary, we have reported an improved method for treating cases of severe PAS based on the relative position of the placenta and hemostatic tools (tourniquet or forceps). Our method resulted in the reduction of intraoperative bleeding, particularly for cases with lower placental implantation. Thus, patients can receive effective and individualized treatment for severe PAS.

## Data Availability Statement

The original contributions presented in the study are included in the article/Supplementary Material, further inquiries can be directed to the corresponding author.

## Ethics Statement

The studies involving human participants were reviewed and approved by Medical Ethics Committee of the Xiangya Hospital Central South University. The patients/participants provided their written informed consent to participate in this study.

## Author Contributions

Conceptualization: JH and WZ. Data curation: JH and SD. Funding acquisition: WZ. Investigation, resources, and writing—original draft: XZ and LL. Methodology: JH, XZ, LL, and SD. Project administration: CP, YZ, RL, WW, YJ, YL, HL, and XW. Supervision: JH, XZ, and LL. Writing—review and editing: WZ. All authors contributed to the article and approved the submitted version.

## Funding

This research was funded by the Science and Technology Project of Hunan Province (2017SK2151, 2017SK1033), the National Key Research and Development Program of China (2016YFC1000206), the National Twelfth-Five Year Research and Development Program of China (2014BAI05B05), Clinical Research Project of Xiangya Hospital Central South University (2013L10), the National Natural Science Foundation of China (81974236, 81571516), and the Major Scientific and Technological Projects for collaborative prevention and control of birth defects in Hunan Province (2019SK1010).

## Conflict of Interest

The authors declare that the research was conducted in the absence of any commercial or financial relationships that could be construed as a potential conflict of interest.

## Publisher's Note

All claims expressed in this article are solely those of the authors and do not necessarily represent those of their affiliated organizations, or those of the publisher, the editors and the reviewers. Any product that may be evaluated in this article, or claim that may be made by its manufacturer, is not guaranteed or endorsed by the publisher.
