# Supplementary material for: Placenta Accreta Spectrum Outcomes Using Tourniquet and Forceps for Vascular Control
Source: Front Med (Lausanne). 2021 Oct 18;8:557678. doi: 10.3389/fmed.2021.557678 (PMC8558214; doi:10.3389/fmed.2021.557678)
Supplement: Supplementary file 2 [file Data_Sheet_1.doc]

Supplementary Material

# Supplementary Figure

**
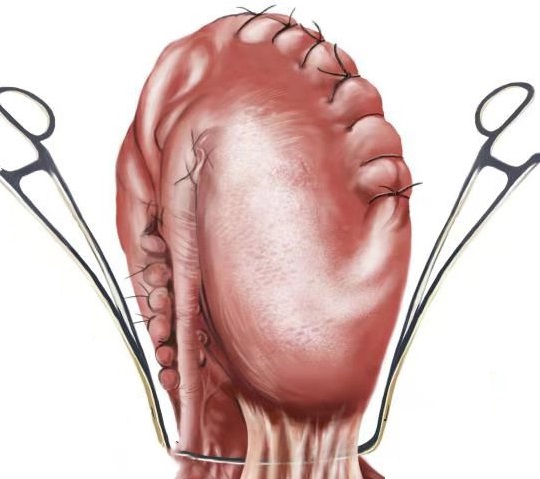
**

**Supplementary Figure 1.** Placement of forceps during surgery. For clarity, the tourniquet is not shown in the figure. The forceps are placed at the level of the external cervix (i.e., top of the vagina) to partially block the placental blood supply.

# Supplementary Video

**Supplementary Video 2.** Video of surgery using tourniquet and forceps. After fetal delivery, a tourniquet was first used in the video to partially reduce the placental blood flow, and the bleeding was reduced after tourniquet application. Then, intraoperative bleeding was further reduced using forceps.
